# Fecobionics characterization of female patients with fecal incontinence

**DOI:** 10.1038/s41598-022-14919-y

**Published:** 2022-06-22

**Authors:** Kaori Futaba, Ssu-Chi Chen, Wing Wa Leung, Cherry Wong, Tony Mak, Simon Ng, Hans Gregersen

**Affiliations:** 1 Department of Surgery, The Chinese University of Hong Kong, Shatin, Hong Kong; 2 California Medical Innovations Institute, 11107 Roselle St., San Diego, CA 92121 USA

**Keywords:** Motility disorders, Gastrointestinal diseases

## Abstract

Defecatory disorders including fecal incontinence (FI) are diagnosed on the symptom pattern supplemented by anorectal manometry (ARM), the balloon expulsion test (BET), and endo-anal ultrasonography. In this study, we used a simulated stool named Fecobionics to study distinct defecation patterns in FI patients using preload-afterload diagrams and to provide comparative data on defecation indices (DIs) between passive and urge incontinent patients. All subjects had Fecobionics, endo-anal ultrasonography and ARM-BET done. The Fecobionics bag was distended in rectum until urge in 37 female patients (64.1 ± 1.5 yrs) and a group of normal subjects (NS, 12F, age 64.8 ± 2.8 yrs). Rear-front pressure (preload-afterload) diagrams and DIs were compared between groups. The FISI score in the patients was 8.6 ± 0.6. The NS did not report FI-related symptoms. All patients and NS defecated Fecobionics and ARM-BET within 2 min. The urge volume was 46.1 ± 3.6 and 35.3 ± 5.9 mL in the FI and normal groups (P > 0.1). The expulsion duration was 14.8 ± 2.4 and 19.8 ± 5.1 s for the two groups (P > 0.1). The preload-afterload diagrams demonstrated clockwise loops that clearly differed between the FI subtypes and NS. The DIs showed profound difference between patients and NS. Fecobionics data showed higher correlation with symptoms in FI patients than ARM-BET. Fecobionics obtained novel pressure signatures in subtypes of FI patients and NS. Fecobionics provides DI data that cannot be obtained with ARM-BET.

## Introduction

Fecal incontinence (FI) is characterized by involuntary loss of rectal content through the anal canal. It is a psychologically and socially debilitating problem that can dramatically affect quality of life and is under-diagnosed. Severely affected FI patients are unable to leave their home, which leads to unemployment, social isolation and depression^1^. 15.3% of the population in USA over 70 years of age and up to 9.5% under 70 suffer from FI^2^. The pathophysiology of this condition is multifactorial. Despite identification of patho-ethiological factors associated with FI such as anal sphincter rupture during vaginal delivery, our understanding can be further improved for adequate treatment of individual patients. FI treatments range from dietary changes to biofeedback therapy and surgery.

The mechanisms of defecation and continence depend on several factors including colorectal motility, stool consistency, rectal capacity and compliance, anorectal sensitivity, and coordination of the pelvic floor muscles and sphincter^3–7^. Management for patients with anorectal functional disorders can be optimized if we obtain a better understanding of the multifactorial control of defecation and continence. Tests for physiological assessment and diagnostics of anorectal disorders are available but may not cover all facets of anorectal function or identify the underlying mechanisms. In particular, the opening characteristics of the anal sphincter complex during incontinent episodes and defecation cannot be described in detail with any currently available exam. Defecography is the only technology that reflects the dynamics of the defecation but unfortunately it does not provide information about anorectal pressures. Furthermore, the balloon expulsion test (BET) assesses the time it takes to defecate the balloon but no other defecatory parameters including pressure^8,9^, and anorectal manometry (ARM) is not done during defecation, though defecation is simulated by the push procedure. Considerable disagreement has been found between the results of various anorectal tests^10^. Furthermore, poor correlation has been found between various tests and with symptoms^11–15^. Current paradigms for defecatory disorders may need a new approach with innovative devices that can provide real time, quantitative, and mechanistic insights by simulating defecation through multi-dimensional measurements.

We are seeking to change the approach to anorectal functional testing with the overall goal to provide mechanistic understanding of defecation using a simulated stool named Fecobionics^16–21^. It integrates BET and ARM. Fecobionics makes it possible to describe the opening characteristics during entry into the relaxing anal canal without disturbing the defecation process. Recently, technological validation^22^ and studies on NS and presumed NS with abnormal ARM-BET^16,23^ were published. It was demonstrated that the axial pressure signatures, preload-afterload analysis, and computation of defecation indices (DIs) with Fecobionics provide useful endpoints^16,22^. Two small-scale feasibility studies indicated that the DIs differed between FI patients, lower anterior resection syndrome (LARS) patients, and NS whereas simple defecatory measures and ARM-BET data did not differ^20,24^.

The primary aim of present study was to study the pathophysiological characteristics and patterns of anorectal function using Fecobionics in female FI patients compared to age-matched female NS. We studied distinct defecation patterns in FI patients using preload-afterload diagrams and provide comparative data on DIs between passive and urge incontinente patients that have not previously been reported. Expulsion characteristics are described with endpoints of physiological and potential clinical value. Furthermore, Fecobionics data are compared to ARM-BET data.

## Methods

### Subjects

**Figure 1:**
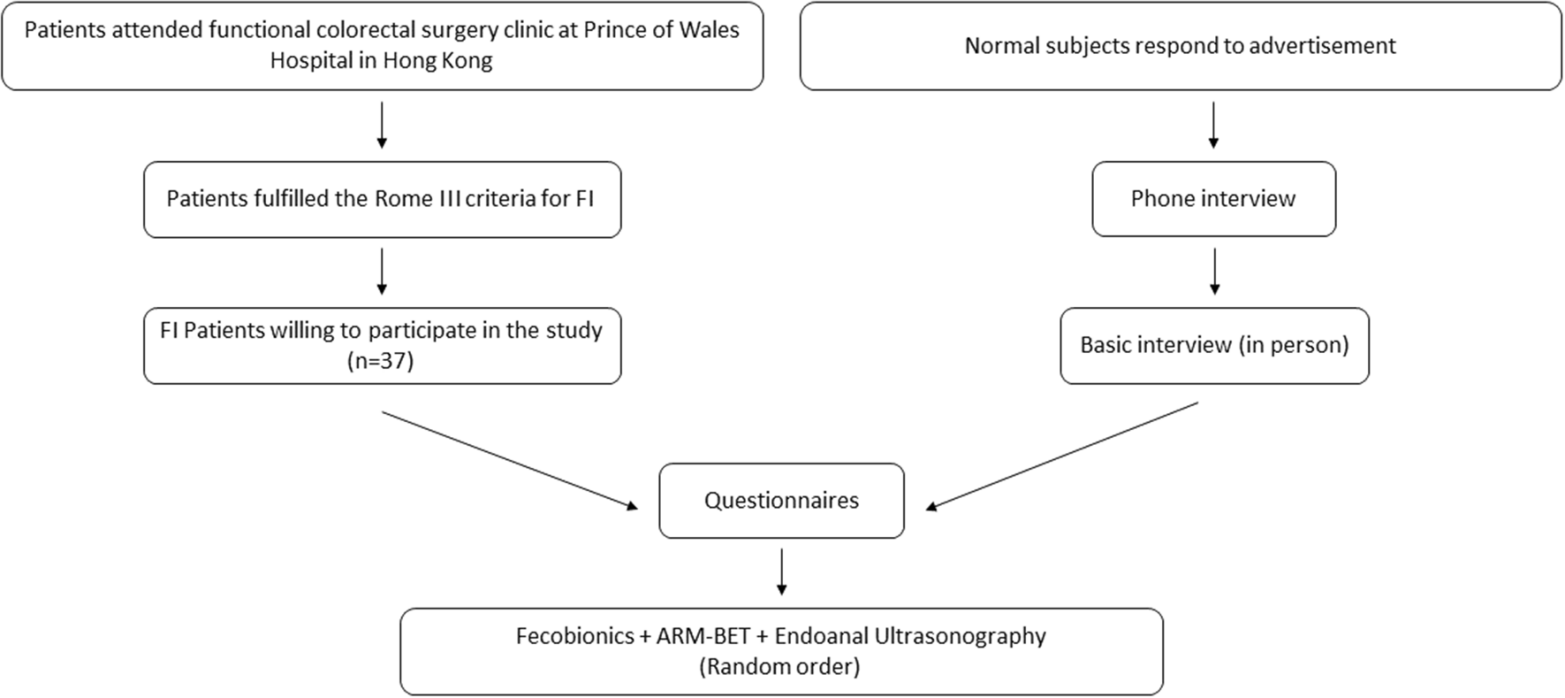
Inclusion and testing scheme.

Thirty-seven patients attending the functional colorectal surgery clinic at Prince of Wales Hospital in Hong Kong were invited to participate in this exploratory study. The patients fulfilled the Rome III criteria for FI^25^. All patients had endo-anal ultrasonography and ARM-BET done. Anal ultrasonography was done to assess damage to the anal sphincters and to measure the anal length. The lower age limit was 18 years. No upper age limit was imposed. Since most of the patients referred to our clinic are women and to avoid gender variation, only females were included. We excluded patients with mixed symptoms to get clearer data for the study. Pregnant women were excluded. Data were obtained on age, health status, body mass index (BMI), symptoms, other diseases and previous treatments. Attention was paid to past medical and surgical history (particularly noting the etiology of FI) and obstetric history. FI Severity Index (FISI) scores and FI QOL scores were obtained^26–28^. The patients were compared to an age-matched group of female NS studied previously with Fecobionics, endo-anal ultrasonography, and ARM-BET. Age-matching (± 1 year) was done from a cohort of normal subjects using a Matlab routine. The inclusion criterion for the control group was asymptomatic normal persons aged over 18 years who gave informed consent. The exclusion criteria were persons with history of chronic constipation or FI, abdominal pain, prior abdominal, pelvic and anal surgery, medication and diseases that may affect bowel function and defecation such as cancer, diabetes and infectious diseases. The subjects were recruited from October 2017 to April 2021. The study scheme is illustrated in Fig. 1.

Prior to functional testing, the subjects were asked to empty their rectum if they were able to. Enema was not used to make the test as natural as possible. Digital rectal examination was performed prior to insertion of Fecobionics to assess anal tone and verify that the lower rectum was empty. Experiments using Fecobionics and ARM-BET were done in randomized order on the same day using a predefined scheme with at least 20 min between the tests. In three cases, the ARM-BET was done on a separate occasion (within 1 week) due to unavailability of the equipment. The London protocol for ARM was followed. All subjects had the tests completed. FISI scores < 5 was considered normal^26^. This study was IRB approved (protocol no. 2017.122, Joint CUHK-NT East Cluster Clinical Research Ethics Committee). All experiments were performed in accordance with relevant guidelines and regulations. All authors had access to the study data and reviewed and approved the final manuscript. Patients or the public were not involved in the design, or conduct, or reporting, or dissemination plans of our research. Trial Registration. http://www.clinicaltrials.gov Identifier: NCT03317938. Date of registration: 21/10/2017. Preliminary data from a subset of the subjects have been reported previously^17^.

### Fecobionics

**Figure 2:**
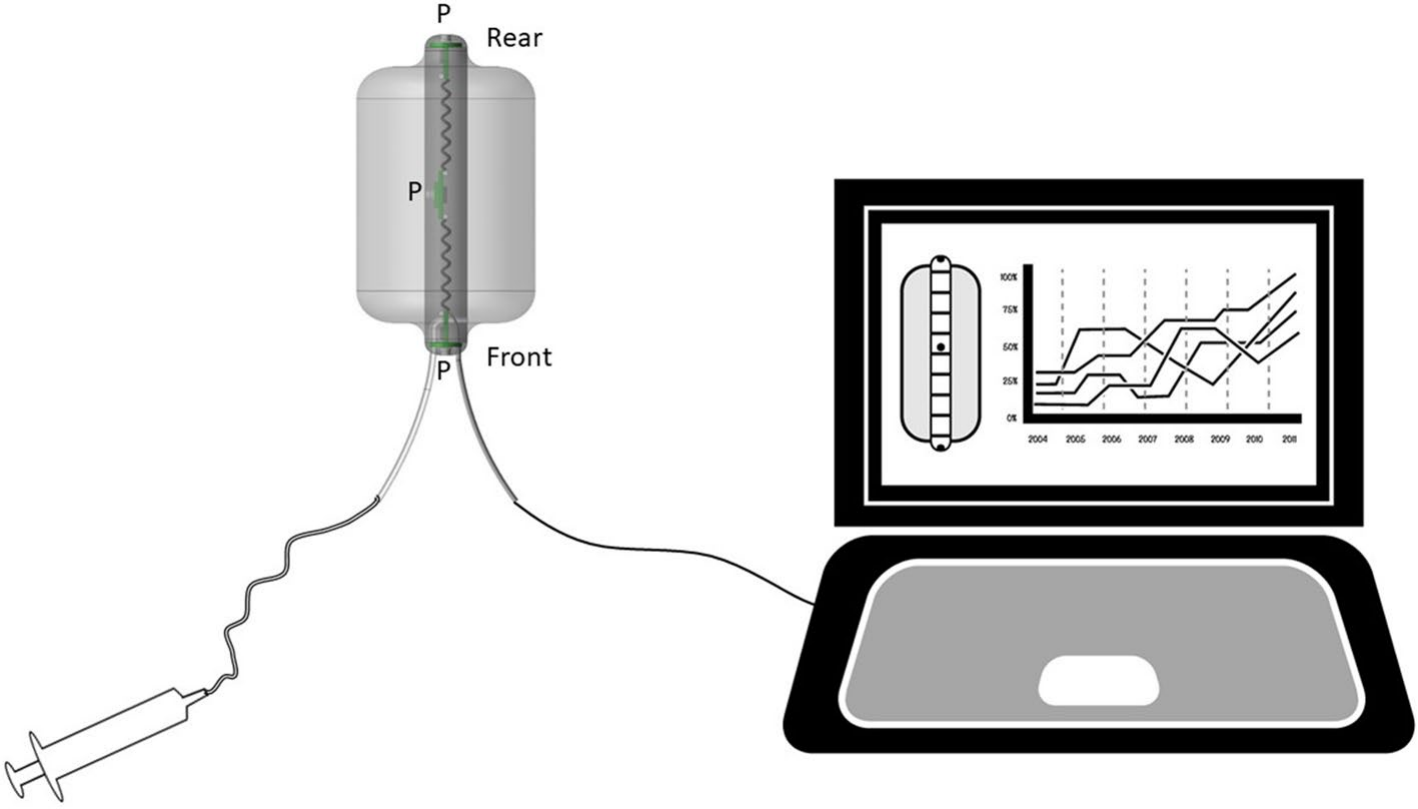
Schematic of the system with the wired Fecobionics device and the data collection device including monitor. The soft resin core of Fecobionics contains the central processing unit and three pressure sensors placed at the front, rear and inside the bag. Furthermore, it contains two Motion Processor Units for determination of bending (not used in this study). Wires and a filling tube is attached at the front. P = pressure sensor.

The basic design of Fecobionics has been described^22,23^ (Fig. 2). Fecobionics was 12-mm-OD, 10-cm-long and made of Silicone rubber (PS6600, Yipin Mould Material Ltd, China). It contained pressure sensors and electronic circuit boards. Miniature pressure sensors (MS5837-30BA, TE connectivity, USA) were embedded in the silicone rubber core at the front, inside the bag, and at the rear of the core. The front and rear sensors pointed in the direction of the defecatory trajectory.

A 30 μm-thick and 8 cm-long polyester-urethane bag spanned most of the core length. The spherically shaped bag contained up to 80 mL without being stretched and had a maximum diameter of 6 cm. The bag was connected through a thin tube extending from the front of Fecobionics to a syringe containing saline.

With the architecture, silicone hardness shore (A5) and the bag, Fecobionics obtained shape and consistency that corresponds approximately to type 4 (range 3–4) on the Bristol stool form scale^29^. The range from types 3–4 is found in + 60% of NS^29^. Wires were threaded inside a thin tube extending from the front to a PCs USB port for power supply and real-time data transmission and display of data. Further processing was done in Matlab.

### Procedures

The settings were made as private as possible using a curtain to shield the patient. Fecobionics was manually inserted into the rectum where after the subjects moved from the bed to the commode chair. After approximately five minutes resting, the subjects were asked to squeeze the anal muscle twice and to cough twice to validate correct placement of Fecobionics. The 5-s-long anal squeezes were done to test to what degree the subjects could contract the anal sphincter. They were asked to refrain from contracting the abdominal muscles at the same time. Afterwards the bag was distended until urge sensation. The urge volume was noted and the subjects were asked to evacuate Fecobionics as they normally do at home and without excessive straining. The investigators left the room and the subjects defecated the device in privacy.

The Fecobionics devices were inspected for leaks and damage or malfunction. Any safety issue and adverse effects were characterized and reported as unanticipated adverse device effects. The subjects were instructed to contact a specific member of the research team if they experienced any problem after leaving the clinic.

ARM-BET was conducted with a standard HRM single-use 8ch anorectal catheter (G-90150, MMS, Enschede, Netherlands). It was inserted with the subjects lying in left lateral position with bended hip and knees. The bag was placed in the rectum and pressure was measured at 0.5 cm distance in the anal canal. Resting anal pressure, maximum anal squeeze pressure, the recto-anal inhibitory reflex (RAIR), urge volume, maximum tolerable volume, and expulsion duration for the 50 mL balloon were evaluated. The ARM system did not provide measure of the rectoanal pressure gradient. BET was done on the commode chair and the investigators left the room during BET defecation.

### Data analysis

Multiple parameters were calculated including the questionnaires score, duration of the Fecobionics experiment, expulsion duration, pressure amplitudes from the rear, bag and front sensors, and the difference between the rear and front pressure sensors (delta pressure, a measure of the rectoanal pressure gradient).

Advanced analyses included the rear-front (preload-afterload) diagrams^3,16,22,30^. The front pressure was plotted as function of the rear pressure as a proxy of the well-known preload-afterload diagrams in cardiology^3,16,22,30,31^. The preload-afterload analogy for defecation is that rectal or abdominal muscle contractions generate the preload whereas the afterload is due to anal resistance. Fecobionics measures the preload and afterload with the rear and front pressure sensors.

**Figure 3:**
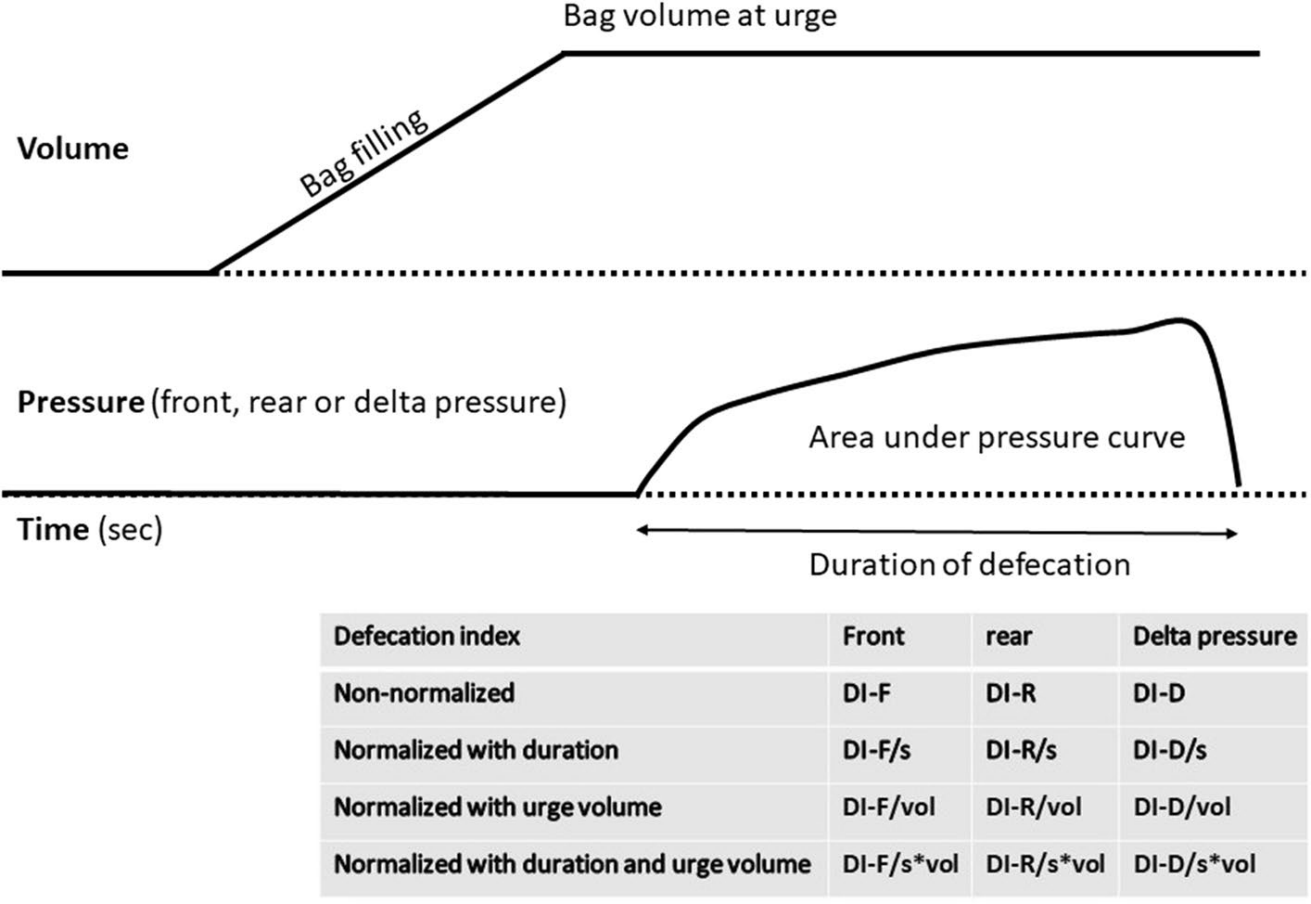
Schematic of the volume and pressure changes during bag filling and defecation of Fecobionics to ease the understanding of the defecation indices (DIs). The area under curve of either the front, rear and delta pressure was computed. They were normalized with respect to defecation duration and urge volume.

Since previous studies have demonstrated wide variability in anorectal parameters and lack of difference between FI patients and normal subjects for simple measures such as the BET expulsion time^17^, we developed several Defecation Indices (DIs). The factors included in the calculation of the DIs are shown in Fig. 3. Basically, the DIs are the areas (integration) of the pressures curves from the rear (R) or front (F) pressure sensors, or from the delta pressure (D). In other words, these DIs represent the accumulated propulsive and resistive loads during defecation. The DIs were normalized with respect to the urge volume and the duration of defecation on an exploratory basis to determine the best parameters for clinical evaluation. For example, DI-R/s*vol was computed as the area under curve of the rear pressure per time unit (sec) and bag volume at urge (mL). This provides 12 DIs as shown in Fig. 3. Furthermore, the defecatory resistance was computed as the maximum pressure difference divided by the urge volume (dP/vol). It is a proxy of the resistance to flow in the anal canal and the reciprocal of the contraction work^30^. Moreover, the DI*vol-R/F ratio was computed.

### Statistics

The sample sizes were largely decided on an exploratory basis. To obtain a significant number of patients, we decided to include at least 35 subjects. For NS, data variation for expulsion duration and volume were available from a previous study^16^. Inclusion of 12 NS was considered sufficient.

Shapiro–Wilk normality test was used to test if data were normal distributed. For parametric data, mean ± SEM were computed and t-test, paired t test and one-way ANOVA were used for studying differences. Median and quartiles and non-parametric statistics including Mann–Whitney' U test and Kruskal–Wallis test were used for non-parametric data. Pearson´s correlation was used for analysis of association of data obtained with the technologies employed. Results were considered statistically significant when *P* < 0.05 (2-tailed). SPSS (v20.0, IBM, New York, USA) and Excel were used for statistical testing.

## Results

All subjects were female Asians living in Hong Kong. Thirty-seven female FI patients and 12 age-matched female NS were included. The patients had repair of uterogenital prolapse (n = 2), rectovaginal fistula with fistulectomy (n = 1), hysterectomy with anterior and posterior repair ± bilateral salpingo-oophorectomy (n = 3), lap loop colostomy with repair of the anal sphincter (n = 1), left-side hemicolectomy (n = 1), perianal repair (n = 2), and Delorme's surgery (n = 1). The normal subjects were asked about obstetric history but did not report specific problems related to pregnancy or birth.

The demographic data did not differ between groups (Table 1). The FISI score was 8.6 ± 0.6 in patients whereas the NS did not report symptoms (Table 1). Of the 37 FI patients, 22 were characterized clinically as having urge incontinence and 15 as passive incontinence. Difference in FISI score was not found between urge and passive FI subtypes (urge: 8.3 ± 0.7 and passive: 9.0 ± 1.0, P > 0.5). The anal length in FI patients and NS was 2.5 ± 0.1 and 2.7 ± 0.2 cm (P > 0.5). Three patient had minor anal sphincter defects visible on endo-anal ultrasonography. Nine patients developed FI after giving birth. Three FI patients did not have RAIR in the ARM evaluation. All NS presented normal RAIR in the ARM evaluation. The digital exploration gave impression of low sphincter tone in most FI patients.

**Table 1:** Demographics, fecal incontinence severity index (FISI) score, Fecobionics, anorectal manometry (ARM) and balloon expulsion test (BET) data for fecal incontinence (FI) patients and normal subjects (NS).

	FI group	NS group	Statistics
**Demographics and FISI score**			
Number of subjects	37	12	n/a
Age (years)	64.1 ± 1.5	64.8 ± 2.8	P > 0.5
Weight (kg)	60.9 ± 1.5	56.6 ± 2.8 kg	P > 0.2
Height (m)	1.6 ± 0.0	1.6 ± 0.0 m	P > 0.5
BMI (kg/m^2^)	24.9 ± 0.6	23.0 ± 1.2 kg	P > 0.2
FISI score	8.6 ± 0.6	0.0 ± 0.0	P < 0.01
Disease duration (years)	3.7 ± 0.7	n/a	n/a
**Fecobionics**			
Anal resting pressure (cmH_2_O)	22.0 ± 2.5	33.9 ± 4.9	P < 0.05
Max squeeze pressure (cmH_2_O)	66.8 ± 4.4	111.9 ± 7.6	P < 0.001
Max defecation pressure (cmH_2_O)	110.8 ± 7.1	129.2 ± 8.8	P > 0.1
Expulsion duration (sec)	11 (4–20)	11 (6–34)	P > 0.5
Urge volume (mL)	46.1 ± 3.7	35.3 ± 5.9	P > 0.2
**ARM-BET**			
Anal resting pressure (cmH_2_O)	65 (48–86)	73 (56–85)	P > 0.5
Max squeeze pressure (cmH_2_O)	199.1 ± 13.2	310.7 ± 23.7	P < 0.001
Expulsion duration (sec)	27 (14–45)	14 (8–22)	P < 0.05
Urge volume (mL)	75 (63–100)	78 (66–98)	P > 0.5
Max tolerable volume (mL)	121.1 ± 5.7	133.9 ± 11.9	P > 0.2

Data in parentheses are quartiles.

### Fecobionics studies in FI patients and normal subjects

None of the Fecobionics studies lasted more than 10 min from insertion to evacuation of the device. No adverse effects were reported during insertion, rectal distension or during evacuation.

Atmospheric pressure levels at the front was measured in two patients when moving to the commode chair. This indicated that the front of Fecobionics was either at a location in the anorectum connected with an air passageway to the outside or was slightly outside anus. The data from these patients were included in the analysis as it would reflect normal conditions in FI patients. One patient dropped (evacuated) Fecobionics when moved to the commode chair. For this patient we only used the ARM-BET, endo-anal ultrasonography, and FISI data.

#### Pre-distension data

The anal resting pressure in the patients and NS was 22.0 ± 2.5 and 33.9 ± 4.9 cmH_2_O (P < 0.05, Table 1). Anal squeezes resulted in pressure increase in the front pressure sensor. The anal squeeze pressure in the patients and NS was 66.8 ± 4.4 and 111.9 ± 7.6 cmH_2_O (P < 0.001, Table 1). The anal resting pressure and the squeeze pressure measured by Fecobionics were lower than the same pressures measured by ARM (P < 0.001). ARM-BET showed lower maximum squeeze pressure in FI patients compared to NS (199.14 ± 13.24 and 310.67 ± 23.70 cmH_2_O, P < 0.001, Table 1). Resting anal pressure measured by ARM did not differ between FI patients and NS. (P > 0.1, Table 1). Coughing induced simultaneous pressure increase in all three Fecobionics pressure channels (typically 100–150 cmH_2_O). In six patients, the squeeze and cough procedures resulted in afterwards drop of the pressure to zero or near-zero. It indicates the presence of an air passageway to the outside or that the tip was slightly outside anus. However, the bag pressure sensor did not indicate that the bag had moved into the anal canal. The data from these patients were included in further analysis since it reflects normal conditions in these patients. Difference was found in Fecobionics anal squeeze pressure between NS (111.9 ± 7.6 cmH_2_O) and the two FI subtypes (urge: 70.1 ± 6.3 cmH_2_O, P < 0.001; passive: 61.8 ± 5.0 cmH_2_O, P < 0.001). Difference was found in ARM-BET squeeze pressure between the urge and passive FI subgroups (urge: 222.7 ± 16.9 and passive: 164.6 ± 18.0 cmH_2_O, P < 0.05, supplementary Table S1).

The anal length in the FI group correlated with Fecobionics squeeze pressure (r = 0.435, P < 0.05). The anal length in the passive group showed high correlation with Fecobionics anal resting pressure (r = 0.876, P < 0.05) and anal squeeze pressure (r = 0.714, P < 0.005). The duration of FI in the passive group showed good correlation with the Fecobionics anal resting pressure (r = − 0.657, P < 0.05). Correlations for these parameters were not found for ARM-BET.

#### Fecobionics bag distension

The bag was slowly distended until the subjects felt urge. The urge volume was 46.1 ± 3.7 and 35.3 ± 5.9 mL in FI patients and NS (P > 0.2, Table 1). Four of the 37 FI patients (11%) reached the 80 mL max bag volume before feeling urge. One of the 12 NS reached the 80 mL volume (8%). The urge volume in patients and NS was not associated with the weight, BMI or age of the subjects (P > 0.5). The urge volume measured by Fecobionics was significantly lower than the ARM-BET urge volume (P < 0.001). The volumes by the two methods were not associated (r = 0.299, P > 0.05). In one patient, the bag filling resulted in front pressure drop to atmospheric pressure. Overall, the ten patients with the front pressure dropping to near-zero before or during bag filling, all had low ARM resting pressure (35.6 ± 6.7 cmH_2_O) compared to the rest (56.2 ± 4.2 cmH_2_O) (P < 0.05). Difference was not found between the NS and the two FI subtypes as measured both by Fecobionics and ARM-BET.

The FISI score did not correlate significantly with any simple distension parameters for Fecobionics (r = − 0.034, P > 0.5) and ARM-BET (r = 0.017, P > 0.5). The anal length in the passive group correlated with the ARM-BET max tolerable volume (r = 0.682, P < 0.05).

#### Evacuation of Fecobionics

**Figure 4:**
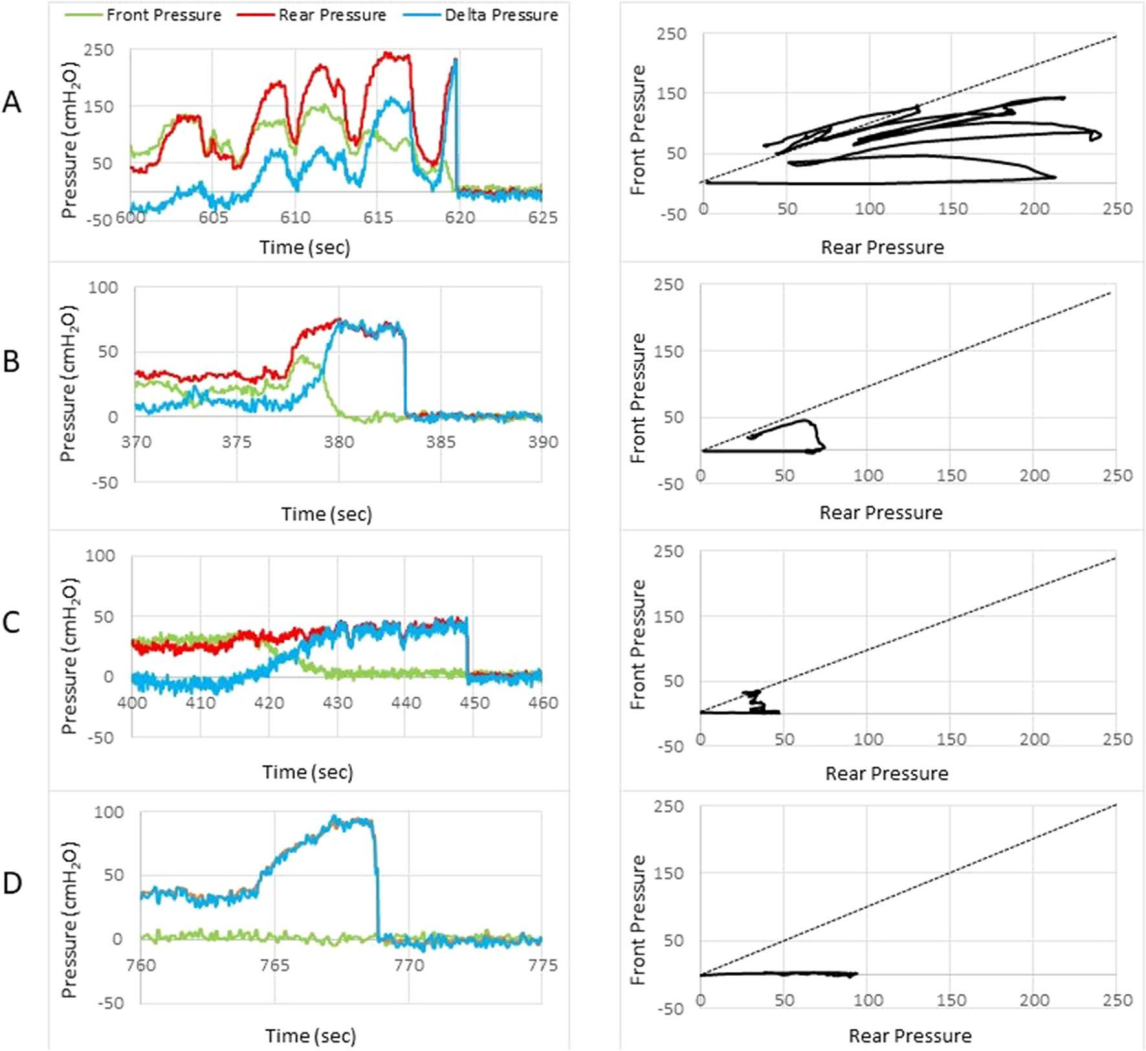
Representative examples of defecations from three FI patients and a normal subject. The left diagrams illustrate the front and rear pressures and the delta pressure as function of time. The right diagrams show the front pressure as function of the rear pressure. The stippled line is the line of unity (front pressure equals rear pressure). The patients defecated Fecobionics with one contraction whereas the normal subject used five contractions. A large variety was found in FI patients where some patients appeared like normal subjects. Others had very low anal pressure.

**Figure 5:**
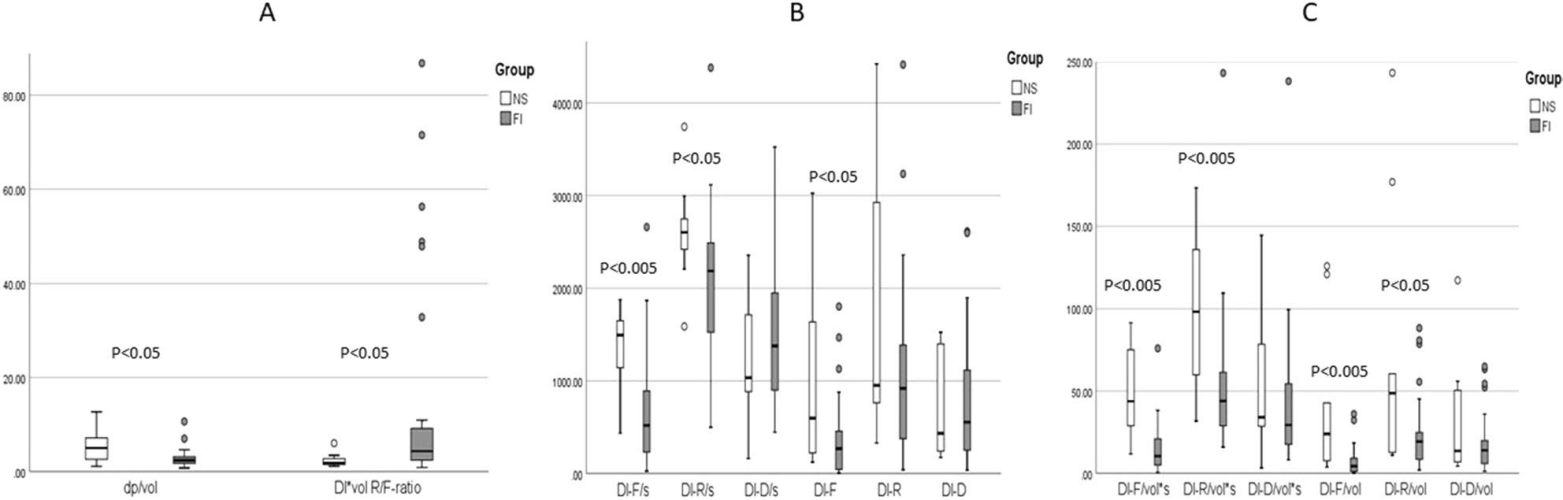
The three diagrams show comparative data of the Defecation Indices (DI). Most Defecation Indices show difference between normal subjects and FI patients. The box represents the interquartile range, which contains the middle 50% of the records. The line across the box indicates the median. The whiskers are lines that extend from the upper and lower edge of the box to highest and lowest values which are no greater than 1.5 times the IQ range. Outliers are cases with values between 1.5 and 3 times the IQ range, i.e., beyond the whiskers. See text for statistical values. F, R and D are the front, rear and delta pressure measurement, vol = volume and s = seconds.

After the urge sensation or maximum volume was reached, the subjects were asked to evacuate Fecobionics. All FI patients and NS defecated Fecobionics. None of the FI patients and NS reported pain or other symptoms during the procedures. Bleeding from the anus was not observed post-defecation. Evacuation data are depicted in Figs. 4 and 5.

The maximum defecation pressure difference between the rear and front sensor (rectoanal pressure gradient) was 110.8 ± 7.1 and 129.2 ± 8.8 cmH_2_O (P > 0.1, Table 1) in FI patients and NS. The expulsion duration was 14.8 ± 2.4 and 19.8 ± 5.1 s for the FI patients and NS (P > 0.1, Table 1). The median BET expulsion duration was longer in FI patients (27, quartiles 14–45) than in NS (14, quartiles 8–22) (P < 0.05, Table 1). The expulsion duration differed in the FI group for Fecobionics vs ARM-BET (P < 0.001). The expulsion duration by the two methods showed positive correlation in the FI group (r = 0.429, P < 0.01). The FISI score showed poor correlation with expulsion duration measured by Fecobionics (r = 0.002, P > 0.5) and ARM-BET (r = 0.007, P > 0.5). For the expulsion duration, no difference was found between the urge and passive FI subgroups (Supplementary Table S1) or between NS and the two FI subtypes.

Figure 4 shows representative patterns of evacuations from a normal subject and three FI patients. The preload-afterload diagrams express clockwise contraction cycles (Fig. 4, right). In most subjects, the first part of the tracing followed the slope of the unity line. All NS were above or on the unity line, whereas 22 of the FI patients were below. The 37 FI patients fell into three groups based on distinct defecation patterns.

*Group 1* Subjects (n = 21, 11 urge and 10 passive, Fig. 4B) showed a somewhat similar pattern as in NS (Fig. 4A) but in general with much lower pressures. The initial increase in the front pressure soon leveled off, indicating anal sphincter relaxation and movement of the front of Fecobionics into the anal canal. The rear pressure usually peaked after the front measured atmospheric pressure. The most significant differences between FI and NS were the lower pressures and the loops below the line of unity in the FI patients compared to NS.

*Group 2* Subjects (n = 6, all urge, Fig. 4C) was characterized by a very low contraction pressure, only one defecatory contraction, and fast decrease of the front pressure to zero. This pattern was not found in any of the NS or passive FI.

*Group 3* Subjects (n = 9, 5 urge and 4 passive, Fig. 4D) was characterized by very low or zero pressure in the front pressure sensor. This indicates that the sphincter was very weak, an air passageway was present, or the front sensor was outside anus from the beginning of the evacuation. Three patients (2 urge and 1 passive) in this group had anal sphincter defect visible on endo-anal ultrasonography.

#### Fecobionics defecation indices

All DIs are shown in Fig. 5. In contrast to the expulsion duration and bag volume, the DIs were all lower in FI patients than in NS, e.g. median DI-F/s (519, quartiles 230–885) compared to NS (1463, quartiles 1183–1637). Statistical difference was found for DI-F/s (P < 0.005), DI-R/s (P < 0.05) and DI-F (P < 0.05), whereas no statistical difference was found for DI-R (P > 0.1, Fig. 5B), DI-D/s and DI-D (P > 0.5, Fig. 5B). Difference were also found for DI-F/vol*s (P < 0.005), DI-R/vol*s (P < 0.005), DI-F/vol (P < 0.005) and DI-R/vol (P < 0.05, all Fig. 5C), whereas no statistical difference was found for DI-D/vol*s and DI-D/vol (P > 0.1, Fig. 5C). The subjects with the highest DI values all suffered from urge incontinence. However, other patients with urge incontinence had low values, and pronounced differences for the averages were not found between the urge and passive FI subtypes. Data were in general scattered in the urge patients, indicating that patients with urge represent a wide spectrum or may have several subtypes of urge incontinence. The median defecatory resistance (dp/vol) was smaller in FI patients (2, quartiles 2–3) compared to NS (5, quartiles 3–7) (P < 0.05) whereas the DI*vol-R/F ratio was highest in the patient group (Fig. 5A).

The DIs did not differ between the urge and passive FI subgroups (Supplementary Table S1). However, difference was found between NS and the two subtypes of FI patients for DI-F/s (P < 0.01), DI-F (P < 0.05) and DI-F/vol*s (P < 0.001). Difference was found between the normal group and the passive FI subgroup for dp/vol (P < 0.05), DI-R/s (P < 0.05), DI-R/vol*s (P < 0.005), DI-F/vol (P < 0.01) and DI-R/vol (P < 0.05).

The FISI showed correlation with DI-R/s in the FI group (r = − 0.382, P < 0.05) and urge group (r = 0.474, P < 0.05). Furthermore, the FISI correlated well with DI-D/s in the FI group (r = − 0.389, P < 0.05) and FI urge group (r = 0.454, P < 0.05). The duration of FI correlated with dp/vol, DI-R/vol*s and DI-D/vol*s in the FI group (r = 0.512, P < 0.005; r = 0.582, P < 0.001; and r = 0.632, P < 0.001), and FI urge group (r = 0.542, P < 0.05; r = 0.633, P < 0.005; and r = 0.672, P < 0.005). The anal length of the passive FI group showed high correlation with DI-R/s (r = 0.690, P < 0.05) and DI-R/vol (r = 0.760, P < 0.05).

## Discussion

Fecobionics provides a new bionics concept for studying anorectal physiology. Technological validation, and small-scale feasibility (performance) data in NS, FI patients, LARS patients and constipation patients including modeling data were reported previously^16,17,19–24,32,33^. The present study go beyond the previously published small-scale study in FI patients^17^ with inclusion of 37 female FI patients (22 urge FI and 15 passive FI), description of three distinct patterns in FI patients on preload-afterload diagrams, comparison of urge and passive incontinent patients, as well as optimized analysis. Despite that the patient cohort only had mild FI based on the FISI scores and the exploratory nature of the study, we found profound differences. The aim was to describe defecatory pathophysiology and FI phenotypes to serve as a reference for future larger scale clinical studies with further optimized technology and analysis^17,34,35^. We demonstrated successful access in all subjects with no device-related adverse events. Simple parameters including distension volume and expulsion duration were insufficient to distinguish FI patients from NS. The preload-afterload diagrams and the DIs are preferable since they distinctly differed between groups. The DIs showed higher correlation to the FISI score than ARM-BET parameters.

### Methodological aspects

Though it was not the primary aim of this to study to compare ARM-BET with Fecobionics, we found profound differences for several measures including the expulsion duration and urge volume. However, for most measures, some degree of correlation was found, e.g. low anal pressure measured with Fecobionics was in general associated with low ARM pressure. In brief, Fecobionics showed differences between FI patients and NS for anal resting pressure, anal squeeze pressure, max dP, dp/vol and most DIs. The differences found with ARM-BET were for max squeeze pressure and expulsion duration but surprisingly, since patients with mixed FI and constipation were excluded, the FI patients had longer expulsion duration than the NS. The main conclusions from comparative studies^16,23^ and from this paper are that values differ between technologies. This is not surprising considering the differences in technology and procedures, e.g. Fecobionics measures axial pressures whereas ARM measures radial pressures. Furthermore, Fecobionics is placed in the lower rectum. This may explain why Fecobionics induced urge at much lower volumes both in normal subjects and FI patients compared to ARM. The position in the lower rectum was confirmed by the low front pressures measured in some subjects, which we interpret as the presence of air passageways or a slight descent due to a weak sphincter. Other artificial stool technologies such as BET and the FECOM device merely measure the expulsion duration. Fecobionics has the advantage that it also measures pressures and new metrics can be derived. Due to the differences between technologies, separate normative scales for Fecobionics must be identified.

It is a limitation of the present study that we only assessed urge sensation and that the ARM-based IAPWG protocol could not be followed stringently for Fecobionics. The ARM system did not provide data on the rectoanal pressure gradient, hence this parameter could not be compared. However, it is a general belief that the rectoanal pressure gradient is important for studying obstructed defecation rather than FI. Another potential limitation is that the study was done on an Asian population. However, the subjects were accustomed to defecating in seating position rather than squatting and to the best of our knowledge, no study have shown major differences in anorectal function across human races^21^. Furthermore, the patients had relatively mild FI. Therefore, the results may not be generalizable to populations with more severe FI. Moreover, we excluded patients with mixed FI and constipation symptoms to get clearer data for the study. The implication is that the data may not be generalizable to populations with mixed symptoms. Ideally, the patients should have filled out constipation questionnaires in addition to the FI questionnaire.

### Physiological and pathophysiological aspects

Defecation is a complex physiological process^3,4^. As the fecal mass expands the rectum, stretch receptors stimulate desire-to-defecate^3,31^. The rectum shortens as material is forced into the anal canal and the anal sphincter and puborectalis muscle allow the feces to pass. The evacuation process may easily get disturbed, resulting in symptoms such as pain, FI and constipation^36^. The defecatory process needs to be studied as physiologically as possible. However, the anal opening characteristics due to internal anal sphincter relaxation cannot be studied in detail with current technologies. Fecobionics offers a new paradigm for anorectal diagnostic testing, simulating normal defecation. It is important to develop methods with high correlations to symptoms since several studies showed poor correlation^10,37^. We found that Fecobionics data had higher correlation to symptoms than ARM-BET.

According to the literature^38–40^ and this study, simple measures including expulsion duration and urge volume are insufficient for differentiating between FI patients and NS. Anal resting pressure and squeeze pressure provide relevant assessment of the anal sphincter capability. However, significant additional information can be derived from the clockwise preload-afterload loop diagrams. As previously published in NS^16,23^, repeated expulsion contractions shift the loops downwards. At some point, a threshold is reached where the anal pressure drops quickly followed by expulsion. The preload is generated by rectal or abdominal contraction and afterload is due to anal resistance. It allows evaluation of pressures cycles without the time element where rectum or abdominal muscle contractions generate the preload and the afterload reflects anal resistance. The preload must exceed the afterload before evacuation can take place because defecation cannot occur against a negative rectoanal pressure gradient. Fecobionics (and feces) will be expelled when the recto-anal pressure gradient is large enough to overcome the frictional force between the surface and mucosa. The line of unity pressure is shown in Fig. 4. When the front pressure is higher than the rear pressure, data are above the line of unity. Most FI patient were below the line of unity at all times, which facilitates leakage of rectal contents. In some FI patients (n = 7), the front sensor recorded atmospheric pressure during the move to the commode chair, anal squeezes, cough and bag filling. This indicates that the sphincter is very weak and allows an air passage way into rectum or that the front of the device hang outside anus. Most others expelled the device fast due to higher preload than afterload (normal subjects often prepare for expulsion by running several cycles with gradual changes towards evacuation)^16^. All patients who dropped the device had maximum squeeze pressure below average. Furthermore, most of the patients who dropped the device had resting pressure, urge volume and maximum tolerable volume lower than the average.

The value of the preload-afterload diagrams is convincingly illustrated in Fig. 4 and the data facilitated computation of the DIs. Numerous normalized on non-normalized DIs were developed since this is an exploratory study. It will guide future studies on which DIs to use. The DIs represent the accumulated propulsive, resistive and differential loads during defecation. There were clear differences in DIs between NS and FI. However, they need to be tested in larger studies of FI subtypes, as well as in patients with other anorectal disorders, and may be modified accordingly. In this study, the DIs indicated that several FI subgroups exist even within the urge incontinent group. In this exploratory study, we merely included a broad range of FI patients without paying special attention to the etiology. Pronounced differences were not found between urge and passive incontinent patients, i.e. follow up studies may look into other ways of subtyping FI patients.

Our data point towards compensatory mechanisms in FI patients. It is obvious from biomechanics that low anal resting and squeeze pressures will be associated with low anal resistance. To shed further light on this issue, the dP/vol parameter was computed. It is a measure of resistance to defecation and reciprocal to contraction work. dP/vol was significantly lower in the FI group. If the anal resistance is low, one would expect that patients would use less effort to evacuate. The DIs clearly confirmed this, which points to that the patients compensate when they defecate Fecobionics, i.e. the patients may have developed the habit not to increase the abdominal pressure much to defecate.

FI patients are often divided into urge and passive subtypes by their description and symptoms^41,42^. In general, there is difficulty in stratifying FI patients into subgroups. A review^43^ stated that several anorectal manometry studies for subtypes of FI exist but with discrepancy among results and mixed phenotypes of FI seems to be neglected. Furthermore, classification based on clinical presentation of FI may be oversimplified and symptoms may overlap^44^. At large, we did not find statistically significant differences between the urge and passive FI patients, which is consistent with previous literature^45^. On the other hand, we noticed that all DIs were lowest in the passive group. Therefore, adding more subjects or combining the DIs in a different way may have resulted in statistical difference. Yet we are at an exploratory stage of determining the optimal DIs. This need further study. Furthermore, we stratified the FI patients into three subgroups based on defecatory characteristics and severity as shown in Fig. 4. It is noteworthy that group 2 consisted only of urge patients where the rear pressure only increased marginally before the anal sphincter relaxed, and that group 3 had very low sphincter pressure and often sphincter damage as verified by endo-anal ultrasonography.

It is important to develop methods with high correlations to symptoms since several studies showed poor correlation^10,37^. The DIs showed higher correlation to symptoms than simple parameters measured by Fecobionics and ARM-BET. The DI-R/s showed a negative correlation with FISI score in the FI group, consistent with that patients with high FISI score used lower defecatory work to defecate Fecobionics. Low preload can only result in defecation if the pelvic floor is weak (low afterload).

## Conclusions and future aspects

We demonstrated successful Fecobionics application in FI patients. Fecobionics provides several improvements to current anorectal functional assessment technologies including mechanical properties that mimic stool and pressure measurements in the direction of the trajectory. Profound differences were found between Fecobionics and current technologies. Simple measures including expulsion duration and urge volume are not clinically meaningful. We found that Fecobionics showed higher association with symptom scores than ARM-BET but the clinical significance of this remains to be studied further. We believe Fecobionics is a reliable quantitative tool for diagnostics and for future assessment of the efficacy of therapy in defecation disorders including FI. This paper establishes ranges for NS and FI patients for expulsion duration, urge volume and for the DIs. However, the present study is exploratory. Larger scale studies are required to further explore parameters, assess phenotypes as well as treatment effects. Future studies may take advantage of improved Fecobionics devices that are wireless and capable of measuring the anorectal angle and the bag shape. Furthermore, future studies may examine the efficacy of Fecobionics as a therapeutic tool for performing biofeedback treatment in FI patients. Specifically, we seek to identify novel biomarkers, how these biomarkers can help to predict success or failure of biofeedback therapy, and study association between the Fecobionics data and symptomatic relief in FI patients. These results will address our long-term goal of developing and providing mechanistically-based effective FI treatments.

## Supplementary Information

Supplementary Table S1.

## Data Availability

Access to data can be granted upon reasonable request, which should be directed to the corresponding author.
