# Supplementary material for: Fecobionics characterization of female patients with fecal incontinence
Source: Sci Rep. 2022 Jun 22;12:10602. doi: 10.1038/s41598-022-14919-y (PMC9218093; doi:10.1038/s41598-022-14919-y)
Supplement: Supplementary file 1 — Supplementary Table S1. [file 41598_2022_14919_MOESM1_ESM.docx]

**Supplementary Table S1.**

|  | | **Urge group** | **Passive group** | **Stats** |
| --- | --- | --- | --- | --- |
| **ARM-BET** | |  |  |  |
| Anal resting pressure (cmH_2_O) | | 75 (57-90) | 49 (41-74) | P>0.1 |
| Max squeeze pressure (cmH_2_O) | | 222.8±16.9 | 164.7±18.0 | P<0.05 |
| Expulsion duration (sec) | | 25 (14-44) | 27 (14-44) | P>0.5 |
| Urge volume (ml) | | 79 (71-104) | 70 (57-87) | P>0.2 |
| Max tolerable volume (ml) | | 125.0±8.0 | 115.4±7.6 | P>0.5 |
| **Fecobionics** | |  |  |  |
| Anal resting pressure (cmH_2_O) | | 22.8±3.7 | 20.9±2.9 | P>0.5 |
| Max squeeze pressure (cmH_2_O) | | 70.1±6.3 | 61.8±5.0 | P>0.2 |
| Max defecation pressure (cmH_2_O) | | 115.8±9.8 | 103.3±9.7 | P>0.2 |
| Expulsion duration (sec) | | 13 ( 5-20) | 7 (4-19) | P>0.1 |
| Urge volume (ml) | | 45.0±4.5 | 47.7±6.2 | P>0.5 |
| dp/vol | 3 (2-4) | | 2 (2-3) | P>0.2 |
| DI-F/s | 741.7±151.6 | | 556.3±121.0 | P>0.1 |
| DI-R/s | 2265.4±169.1 | | 1807.5±156.4 | P>0.1 |
| DI-D/s | 1525.3±200.7 | | 1304.1±134.9 | P>0.05 |
| DI-F | 398.2±93.7 | | 282.8±106.0 | P>0.2 |
| DI-R | 1012 (623-1315) | | 456 (344-1303) | P>0.05 |
| DI-D | 853.7±171 | | 580.9±131.8 | P>0.1 |
| DI-F/vol*s | 16.3±3.7 | | 11.7±2.4 | P>0.1 |
| DI-R/vol*s | 52 (33-67) | | 34 (28-54) | P>0.1 |
| DI-D/vol*s | 31 (16-61) | | 24 (18-34) | P>0.05 |
| DI-F/vol | 4 (2-11) | | 3 (1-7) | P>0.1 |
| DI-R/vol | 21 (13-31) | | 16 (7-22) | P>0.1 |
| DI-D/vol | 14 (7-26) | | 8 (5-17) | P>0.05 |
| DI R/F-ratio | 5 (3-6) | | 3 (2-10) | P>0.1 |

**Legend for Supplementary Table S1:** Anorectal manometry (ARM), balloon expulsion test (BET and Fecobionics data for the urge and passive fecal incontinence groups. Data in parentheses are quartiles
